# Supplementary material for: Non-random patterns in the co-occurrence and accumulation of adverse life events in two national panel datasets
Source: Commun Psychol. 2026 Feb 27;4:66. doi: 10.1038/s44271-026-00394-y (PMC13065480; doi:10.1038/s44271-026-00394-y)
Supplement: Supplementary file 2 — Supplementary Materials [file 44271_2026_394_MOESM2_ESM.pdf]

Supplementary Materials
Non-random patterns in the co-occurrence and accumulation of adverse life events in two national panel datasets

Kyra Evers, Denny Borsboom, Eiko Fried, Fred Hasselman, František Bartoš, and Lourens Waldorp

List of Supplementary Figures

S1 Demographics (SHP). 2
S2 Demographics (HILDA). 3
S3 Distribution of the number of observed years per person. 4
S4 Adjusted odds ratios (OR) describing the contemporaneous associations between all adverse life events (SHP). 6
S5 Unadjusted odds ratios (OR) describing the contemporaneous associations between all adverse life events (HILDA). 7
S6 Unadjusted odds ratios (OR) describing the contemporaneous associations between all adverse life events (SHP). 8
S7 Adjusted odds ratios (OR) describing the lag-1 associations between all adverse life events (SHP). 11
S8 Unadjusted odds ratios (OR) describing the lag-1 associations between all adverse life events (HILDA). 12
S9 Unadjusted odds ratios (OR) describing the lag-1 associations between all adverse life events (SHP). 13
S10 Consistency of the distribution of yearly event counts. 14
S11 Contemporaneous joint probability of all event combinations (SHP). 15
S12 Lag-1 joint probability of all event combinations (SHP). 16
S13 Contemporaneous joint probability of all event combinations (HILDA). 17
S14 Lag-1 joint probability of all event combinations (HILDA). 18
S15 Contemporaneous conditional probability of all event combinations (SHP). 19
S16 Lag-1 conditional probability of all event combinations (SHP). 20
S17 Contemporaneous conditional probability of all event combinations (HILDA). 21
S18 Lag-1 conditional probability of all event combinations (HILDA). 22
S19 Range of the accumulation of adverse life events across twenty consecutive years. 24
S20 Fit of heavy-tailed distributions to twenty-year cumulative adverse life event counts. 25

List of Supplementary Tables

S1 Model fit and estimates of contemporaneous associations between events (SHP). 4
S2 Model fit and estimates of contemporaneous associations between events (HILDA). 5
S3 Model fit and estimates of lag-1 associations between events (SHP). 9
S4 Model fit and estimates of lag-1 associations between events (HILDA). 10
S5 Estimates of the Poisson, frailty, and Polya urn models in the accumulation analysis across 10 years. 23
S6 Estimates of the Poisson, frailty, and Polya urn models in the accumulation analysis across 15 years. 23
S7 Model estimates of distributions fit to twenty-year cumulative adverse life event counts. 23

Supplementary Fig. S1: Demographics (SHP).

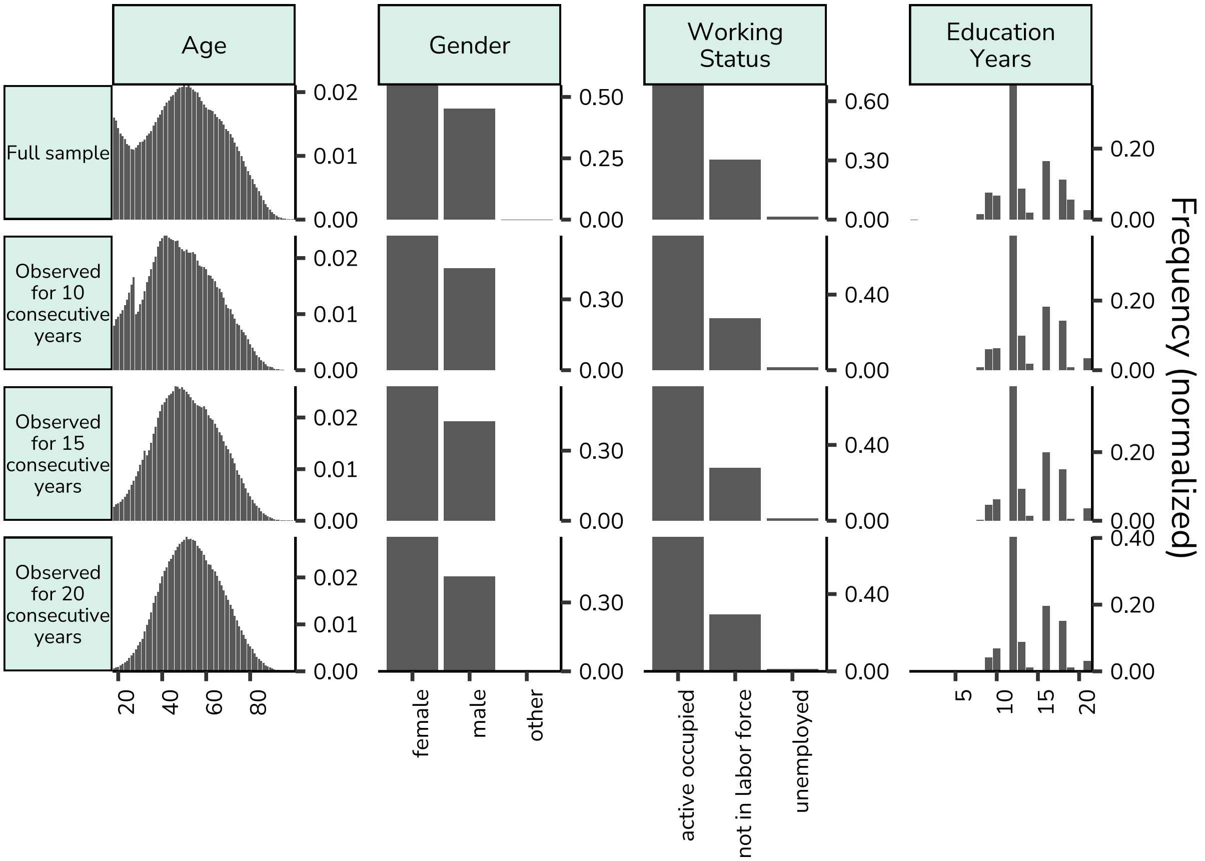

Note. Demographics across person-years based on the overall sample of 27,298 individuals and 15,931 households with 179,310 total person-years (Source: Swiss Household Panel, SHP), as well as the subsamples observed across 10, 15, and 20 consecutive years.

Supplementary Fig. S2: Demographics (HILDA).

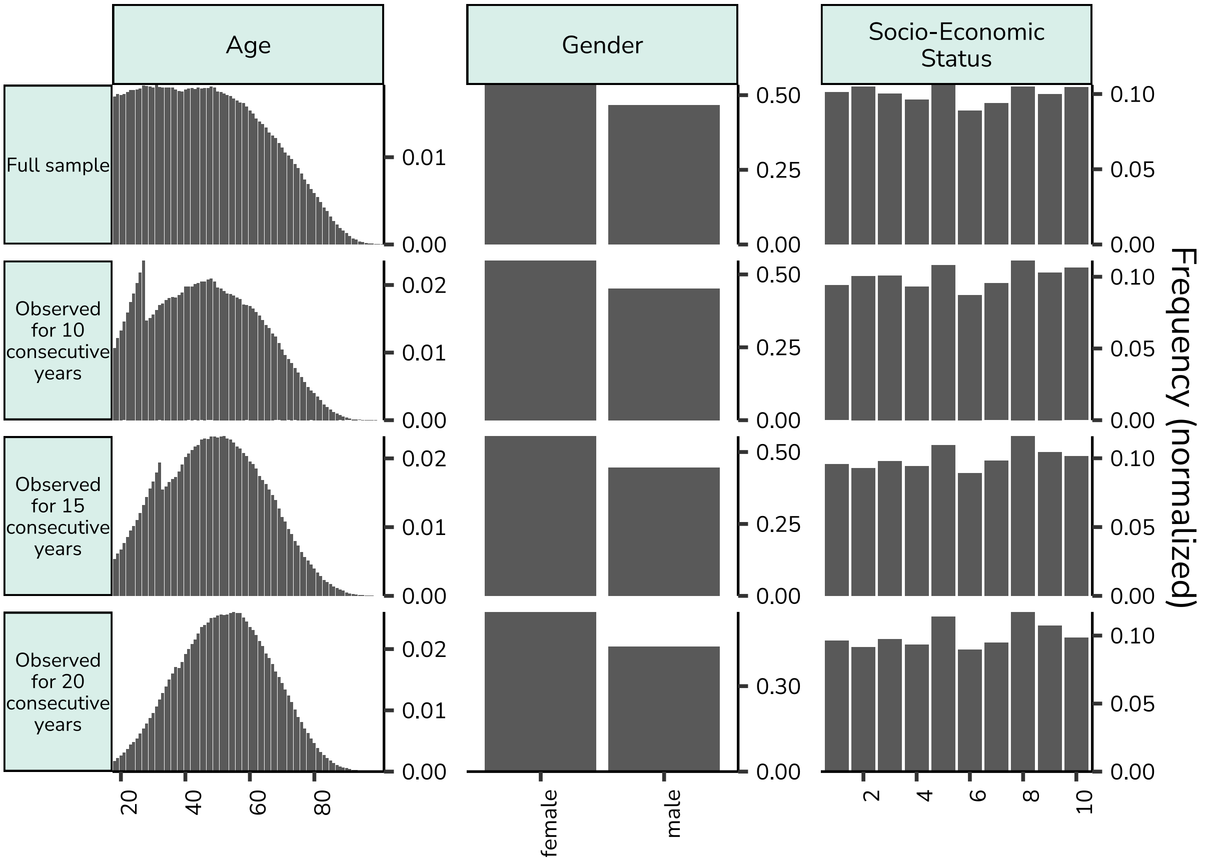

Note. Demographics across person-years based on the overall sample of 30,637 individuals and 37,792 households with 289,083 total person-years (Source: Household, Income and Labour Dynamics in Australia, HILDA), as well as the subsamples observed across 10, 15, and 20 consecutive years.

Supplementary Fig. S3: Distribution of the number of observed years per person.

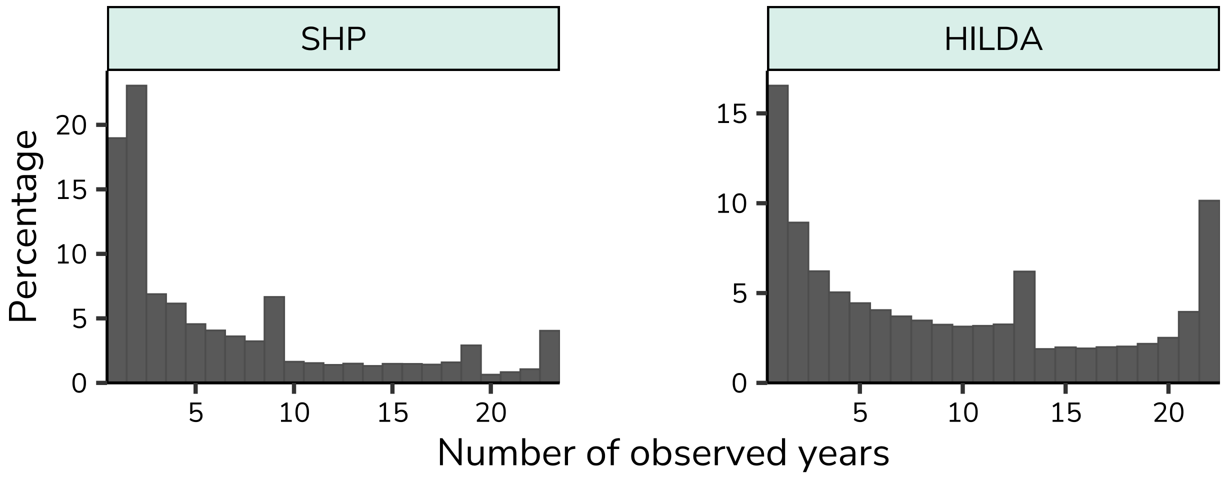

Note. Distribution based on a sample of 27,298 (Source: Swiss Household Panel, SHP) and 30,637 (Source: Household, Income and Labour Dynamics in Australia, HILDA) individuals.

Supplementary Table S1: Model fit and estimates of contemporaneous associations between events (SHP).

	Random σ		Variance Partitioning		
	i	h	Marg. R^2	Cond. R^2	Adj. ICC
Conflicts with or among related persons	1.05 [0.97, 1.12]	0.95 [0.88, 1.03]	0.05	0.41	0.38
Death of closely related person	0.28 [0.24, 0.33]	0.57 [0.54, 0.60]	0.03	0.13	0.11
Termination of close relationship	0.88 [0.78, 1.00]	1.04 [0.95, 1.14]	0.05	0.39	0.36
Accident in home or garden	5.23 [4.76, 5.73]	0.51 [0.04, 7.15]	0.00	0.89	0.89
Illness or accident of closely related person	0.62 [0.59, 0.66]	0.48 [0.44, 0.52]	0.05	0.20	0.16
Mental illness	6.54 [5.93, 7.21]	2.24 [1.47, 3.42]	0.00	0.94	0.94
Physical illness	1.02 [0.96, 1.08]	0.68 [0.61, 0.76]	0.00	0.32	0.31
Problems with own children	0.66 [0.60, 0.71]	0.98 [0.94, 1.03]	0.05	0.34	0.30
Road accident	6.88 [6.21, 7.63]		0.00	0.94	0.94
Sport accident	4.12 [3.68, 4.61]	0.75 [0.29, 1.99]	0.00	0.84	0.84
Other or unspecified illness or accident	0.64 [0.54, 0.75]	1.01 [0.94, 1.08]	0.00	0.30	0.30
Work accident	7.50 [6.77, 8.30]		0.00	0.94	0.94

Note. The household intercept had to be dropped for models with no indicated household σ due to convergence issues. i = Individual; h = Household; Marg. R^2 = Marginal explained variance; Cond. R^2 = Conditional explained variance; Adj. ICC = Adjusted Intra-class Correlation Coefficient. Source: Swiss Household Panel (SHP).

Supplementary Table S2: Model fit and estimates of contemporaneous associations between events (HILDA).

	Random σ		Variance Partitioning		
	i	h	Marg. R^2	Cond. R^2	Adj. ICC
Death of close relative/family member	0.59 [0.56, 0.61]		0.06	0.15	0.09
Death of a close friend	1.01 [0.97, 1.04]	0.99 [0.95, 1.03]	0.02	0.39	0.38
Death of spouse or child	6.03 [5.65, 6.43]		0.01	0.92	0.92
Close family member detained in jail	5.30 [4.96, 5.66]	6.57 [6.22, 6.94]	0.00	0.96	0.96
Fired or made redundant	1.69 [1.59, 1.78]	1.45 [1.35, 1.56]	0.02	0.61	0.60
Serious injury/illness to family member	0.82 [0.79, 0.85]	0.74 [0.71, 0.77]	0.06	0.31	0.27
Serious personal injury/illness	1.21 [1.17, 1.24]	0.58 [0.53, 0.63]	0.03	0.37	0.35
Detained in jail	9.65 [8.73, 10.66]		0.00	0.97	0.97
A weather related disaster damaged or destroyed your home	0.24 [0.06, 0.94]	5.65 [5.36, 5.96]	0.00	0.91	0.91
Separated from spouse	3.55 [3.37, 3.74]	5.75 [5.52, 6.00]	0.00	0.93	0.93
Victim of physical violence	5.60 [5.28, 5.95]	5.92 [5.57, 6.29]	0.01	0.95	0.95
Victim of a property crime	0.88 [0.77, 1.01]	3.21 [2.97, 3.46]	0.01	0.77	0.77
Major worsening in finances	3.13 [2.93, 3.34]	3.68 [3.46, 3.91]	0.02	0.88	0.88

Note. The household intercept had to be dropped for models with no indicated household σ. i = Individual; h = Household; Marg. R^2 = Marginal explained variance; Cond. R^2 = Conditional explained variance; Adj. ICC = Adjusted Intra-class Correlation Coefficient. Source: Household, Income and Labour Dynamics in Australia (HILDA).

Supplementary Fig. S4: Adjusted odds ratios (OR) describing the contemporaneous associations between all adverse life events (SHP).

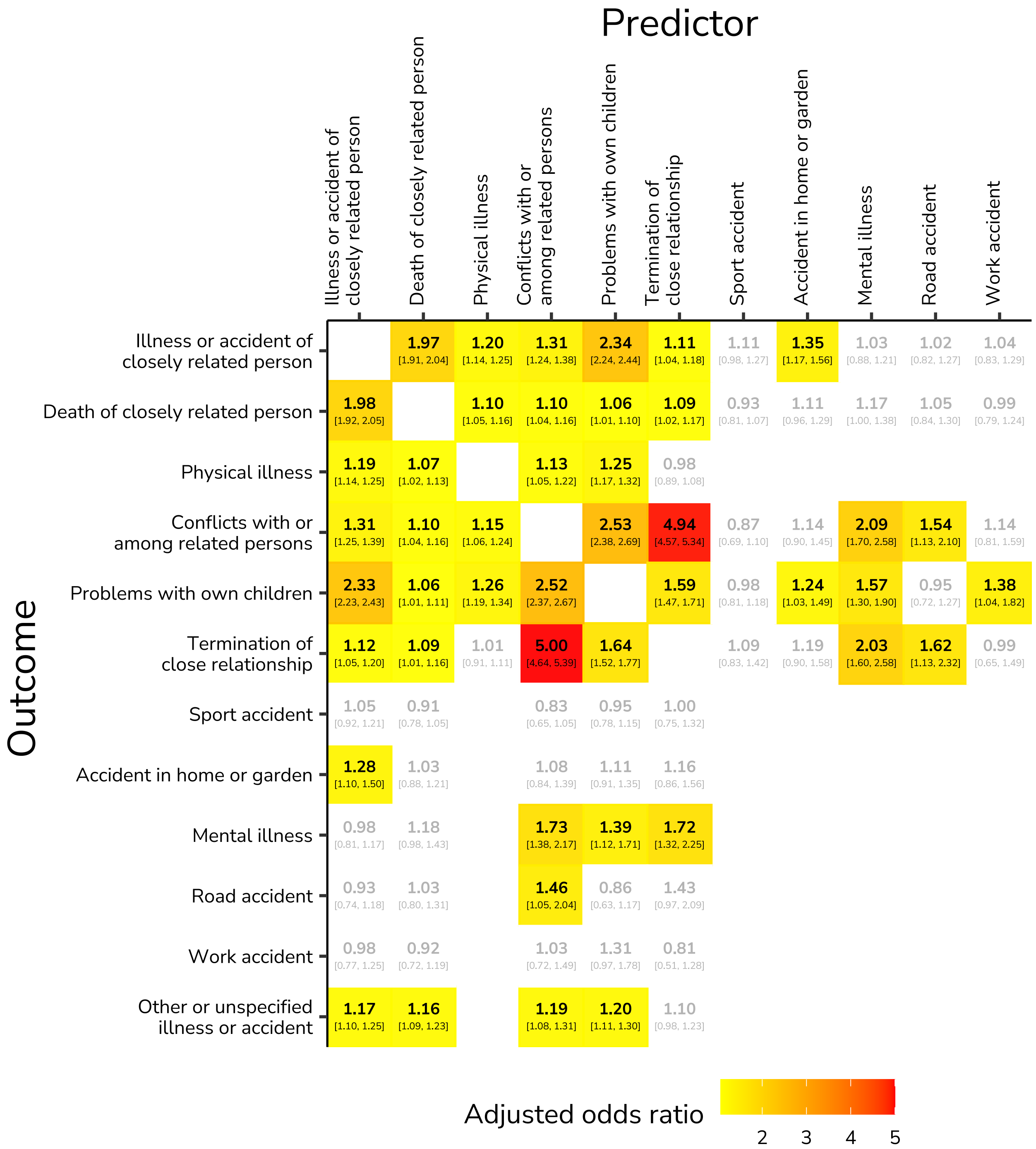

Note. Estimates are based on a sample of 16,946 individuals from 11,231 households with a total of 113,605 person-years (Source: Swiss Household Panel, SHP). OR are adjusted for the effects of other variables, and are coloured from low (yellow) to high (red), which is only shown if the 95% profile confidence interval excluded an OR of one. An OR above one indicates the predictor event increased the odds of the outcome, which was the case for all significant events. Note that different types of personal illness and accidents were mutually exclusive subcategories, such that they could not serve as predictors for events within that larger category. As such, the "other" category was dropped as a predictor to serve as a reference category to avoid collinearity of predictors.

Supplementary Fig. S5: Unadjusted odds ratios (OR) describing the contemporaneous associations between all adverse life events (HILDA).

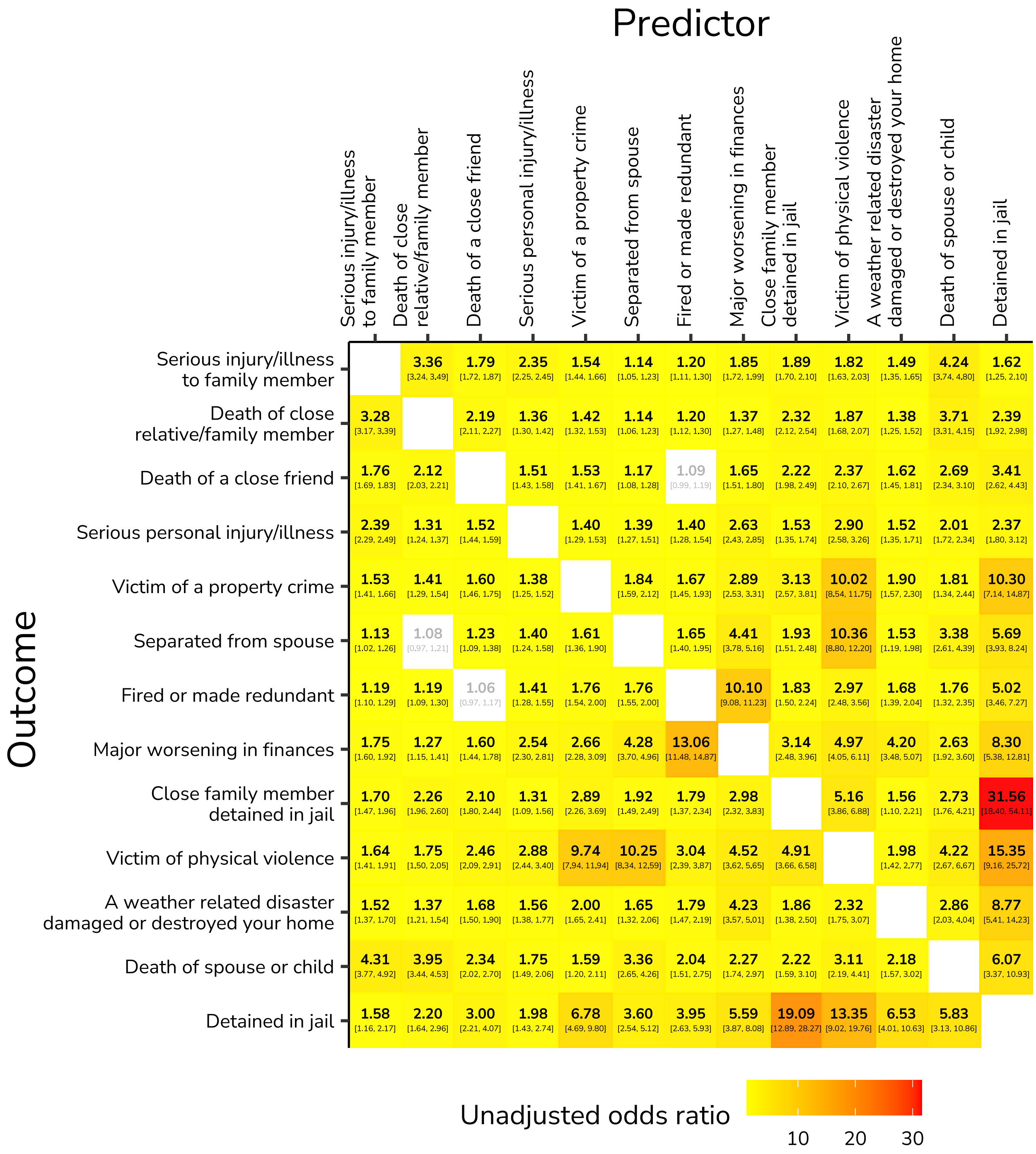

Note. Estimates are based on a sample of 25,803 individuals from 29,410 households with a total of 210,031 person-years (Source: Household, Income and Labour Dynamics in Australia, HILDA). OR are not adjusted for the effects of other variables, and are coloured from low (yellow) to high (red), which is only shown if the 95% profile confidence interval excluded an OR of one. An OR above one indicates the predictor event increased the odds of the outcome event the next year, and an OR below one indicates the odds were reduced.

Supplementary Fig. S6: Unadjusted odds ratios (OR) describing the contemporaneous associations between all adverse life events (SHP).

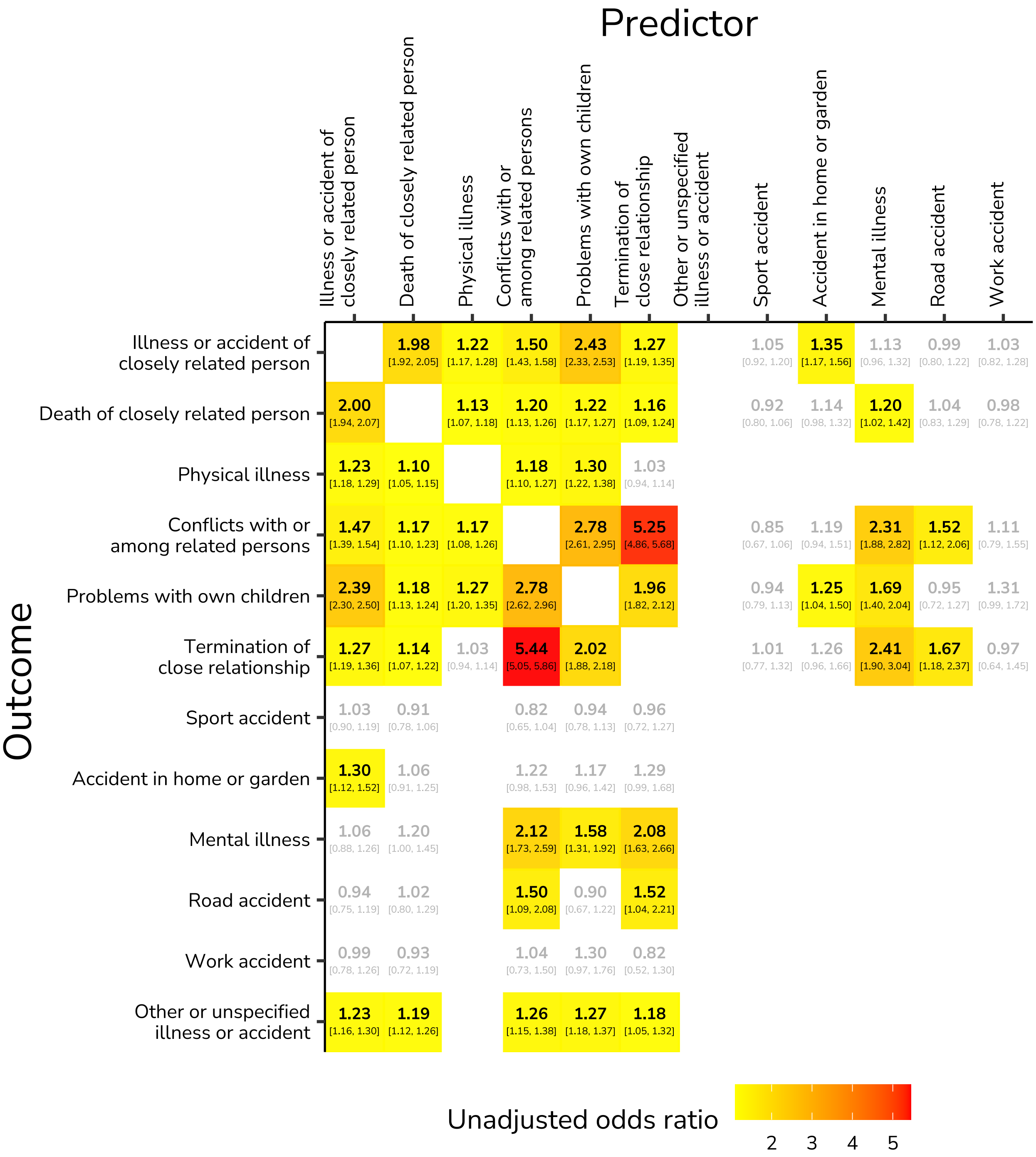

Note. Estimates are based on a sample of 16,946 individuals from 11,231 households with a total of 113,605 person-years (Source: Swiss Household Panel, SHP). OR are not adjusted for the effects of other variables, and are coloured from low (yellow) to high (red), which is only shown if the 95% profile confidence interval excluded an OR of one. An OR above one indicates the predictor event increased the odds of the outcome event the next year, and an OR below one indicates the odds were reduced. Note that different types of personal illness and accidents were mutually exclusive subcategories, such that they could not serve as predictors for events within that larger category. As such, the “other” category was dropped as a predictor to serve as a reference category to avoid collinearity of predictors.

Supplementary Table S3: Model fit and estimates of lag-1 associations between events (SHP).

	Random σ		Variance Partitioning		
	i	h	Marg. R^2	Cond. R^2	Adj. ICC
Conflicts with or among related persons	0.82 [0.74, 0.91]	0.90 [0.83, 0.98]	0.03	0.33	0.31
Death of closely related person	0.16 [0.10, 0.26]	0.51 [0.48, 0.54]	0.02	0.10	0.08
Termination of close relationship	0.55 [0.43, 0.70]	0.99 [0.91, 1.08]	0.03	0.30	0.28
Accident in home or garden	5.02 [4.54, 5.56]		0.00	0.88	0.88
Illness or accident of closely related person	0.51 [0.47, 0.55]	0.44 [0.40, 0.49]	0.03	0.15	0.12
Mental illness	6.43 [5.76, 7.17]	2.08 [1.24, 3.49]	0.00	0.93	0.93
Physical illness	0.71 [0.65, 0.78]	0.49 [0.42, 0.57]	0.04	0.22	0.19
Problems with own children	0.50 [0.44, 0.57]	0.85 [0.80, 0.90]	0.03	0.25	0.23
Road accident	6.90 [6.16, 7.71]		0.00	0.94	0.94
Sport accident	3.53 [2.93, 4.25]	0.76 [0.33, 1.73]	0.00	0.80	0.80
Other or unspecified illness or accident	0.47 [0.36, 0.63]	0.74 [0.65, 0.83]	0.00	0.19	0.19
Work accident	7.54 [6.75, 8.41]		0.00	0.95	0.95

Note. The household intercept had to be dropped for models with no indicated household σ. i = Individual; h = Household; Marg. R^2 = Marginal explained variance; Cond. R^2 = Conditional explained variance; Adj. ICC = Adjusted Intra-class Correlation Coefficient. Source: Swiss Household Panel (SHP).

Supplementary Table S4: Model fit and estimates of lag-1 associations between events (HILDA).

	Random σ		Variance Partitioning		
	i	h	Marg. R^2	Cond. R^2	Adj. ICC
Death of close relative/family member	0.38 [0.34, 0.41]		0.04	0.08	0.04
Death of a close friend	0.82 [0.78, 0.86]	0.87 [0.83, 0.92]	0.02	0.32	0.30
Death of spouse or child	4.85 [4.44, 5.28]	6.12 [5.73, 6.55]	0.00	0.95	0.95
Close family member detained in jail	5.03 [4.66, 5.45]	6.65 [6.25, 7.08]	0.00	0.95	0.95
Fired or made redundant	1.49 [1.39, 1.60]	1.21 [1.11, 1.33]	0.01	0.53	0.53
Serious injury/illness to family member	0.64 [0.61, 0.68]	0.67 [0.64, 0.71]	0.02	0.23	0.21
Serious personal injury/illness	0.94 [0.90, 0.98]	0.49 [0.44, 0.55]	0.03	0.28	0.25
Detained in jail	10.16 [9.01, 11.45]		0.00	0.97	0.97
A weather related disaster damaged or destroyed your home	0.54 [0.35, 0.84]	6.21 [5.88, 6.57]	0.00	0.92	0.92
Separated from spouse	2.88 [2.66, 3.12]	6.17 [5.90, 6.45]	0.00	0.93	0.93
Victim of physical violence	6.25 [5.82, 6.70]	7.10 [6.65, 7.58]	0.00	0.96	0.96
Victim of a property crime	0.87 [0.71, 1.07]	4.06 [3.76, 4.39]	0.00	0.84	0.84
Major worsening in finances	3.60 [3.38, 3.85]	4.57 [4.31, 4.83]	0.00	0.91	0.91

Note. The household intercept had to be dropped for models with no indicated household σ. i = Individual; h = Household; Marg. R^2 = Marginal explained variance; Cond. R^2 = Conditional explained variance; Adj. ICC = Adjusted Intra-class Correlation Coefficient. Source: Household, Income and Labour Dynamics in Australia (HILDA).

Supplementary Fig. S7: Adjusted odds ratios (OR) describing the lag-1 associations between all adverse life events (SHP).

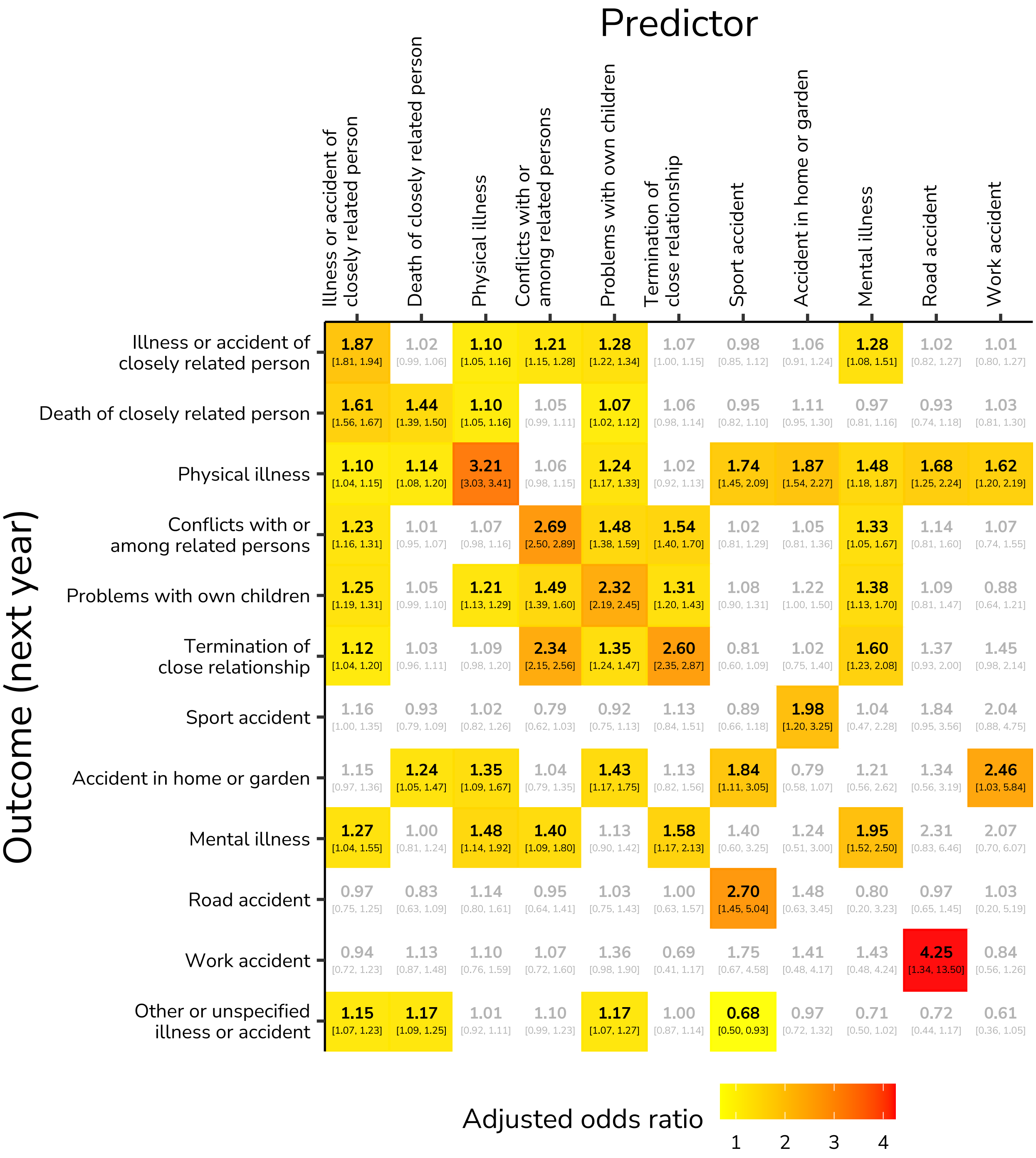

Note. Estimates are based on a median sample of 13,895 individuals from 9,469 households with a total of 92,692 person-years (Source: Swiss Household Panel, SHP). OR are adjusted for the effects of other variables, and are coloured from low (yellow) to high (red), which is only shown if the 95% profile confidence interval excluded an OR of one. An OR above one indicates the predictor event increased the odds of the outcome event the next year, and an OR below one indicates the odds were reduced. Note that different types of personal illness and accidents were mutually exclusive subcategories. As such, the "other" category was dropped as a predictor to serve as a reference category to avoid collinearity of predictors.

Supplementary Fig. S8: Unadjusted odds ratios (OR) describing the lag-1 associations between all adverse life events (HILDA).

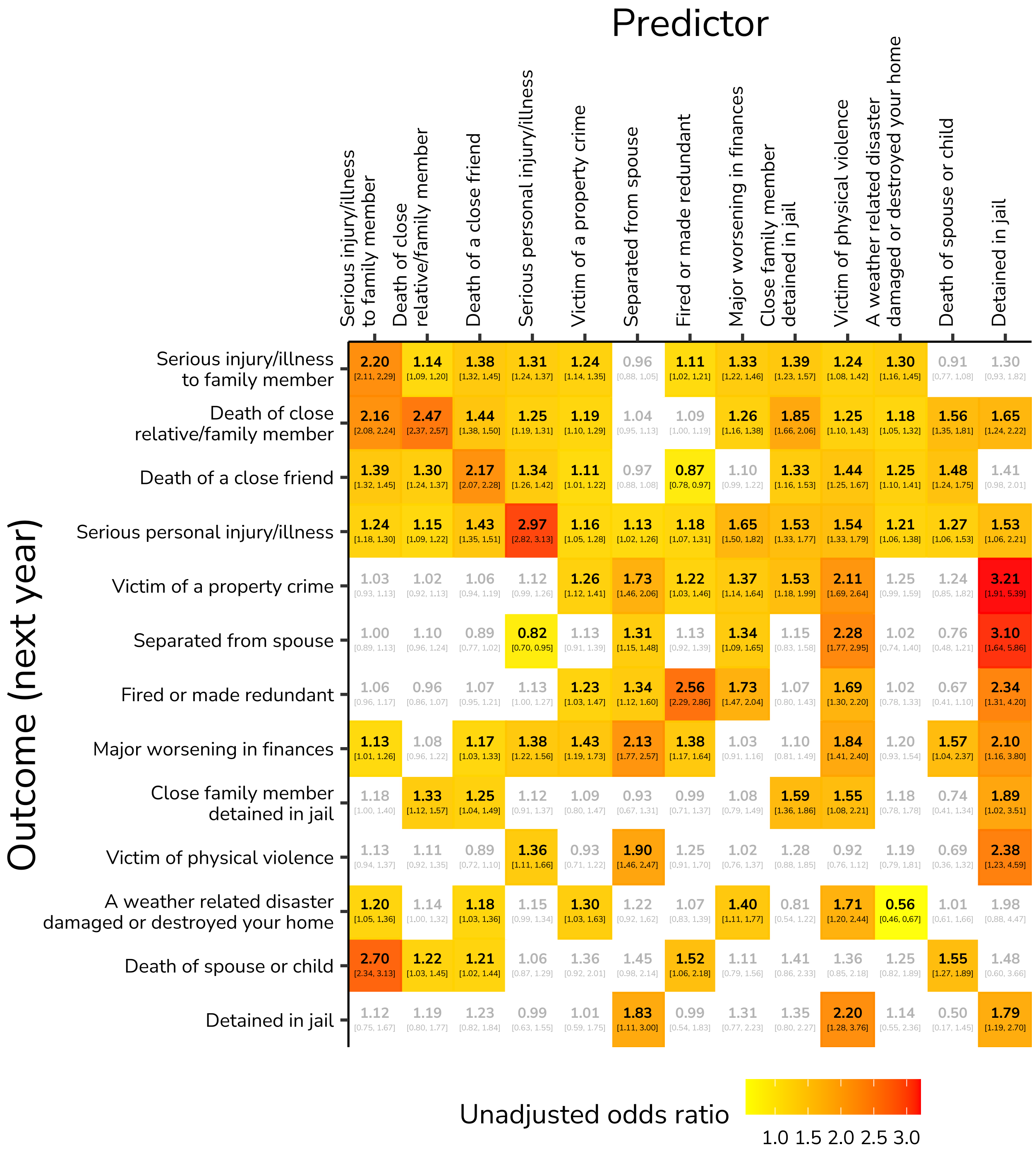

Note. Estimates are based on a median sample of 21,738 individuals from 25,480 households with a total of 174,957 person-years (Source: Household, Income and Labour Dynamics in Australia, HILDA). OR are not adjusted for the effects of other variables, and are coloured from low (yellow) to high (red), which is only shown if the 95% profile confidence interval excluded an OR of one. An OR above one indicates the predictor event increased the odds of the outcome event the next year, and an OR below one indicates the odds were reduced.

Supplementary Fig. S9: Unadjusted odds ratios (OR) describing the lag-1 associations between all adverse life events (SHP).

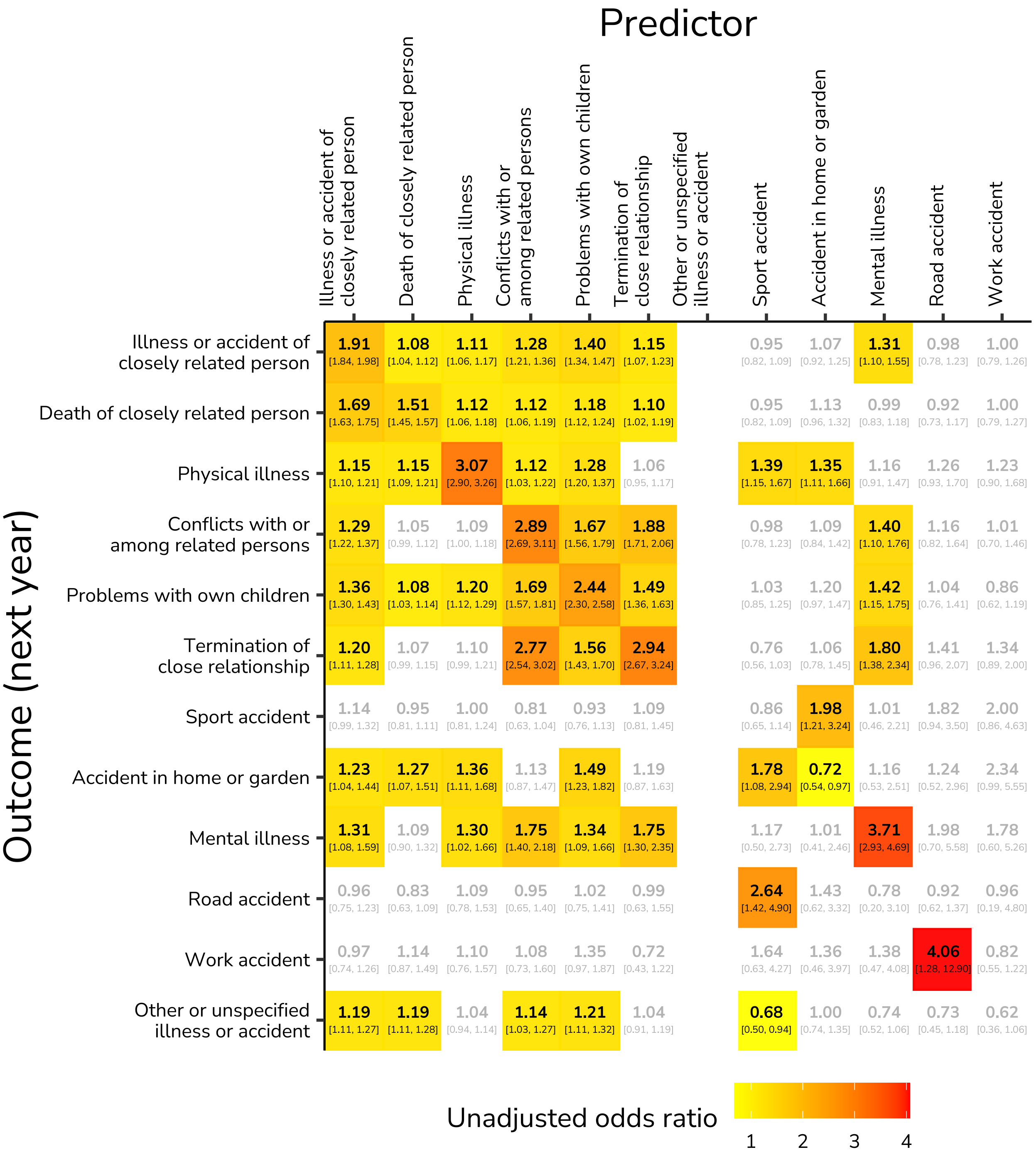

Note. Estimates are based on a median sample of 13,895 individuals from 9,469 households with a total of 92,692 person-years (Source: Swiss Household Panel, SHP). OR are not adjusted for the effects of other variables, and are coloured from low (yellow) to high (red), which is only shown if the 95% profile confidence interval excluded an OR of one. An OR above one indicates the predictor event increased the odds of the outcome event the next year, and an OR below one indicates the odds were reduced. Note that different types of personal illness and accidents were mutually exclusive subcategories. As such, the "other" category was dropped as a predictor to serve as a reference category to avoid collinearity of predictors.

Supplementary Fig. S10: Consistency of the distribution of yearly event counts.

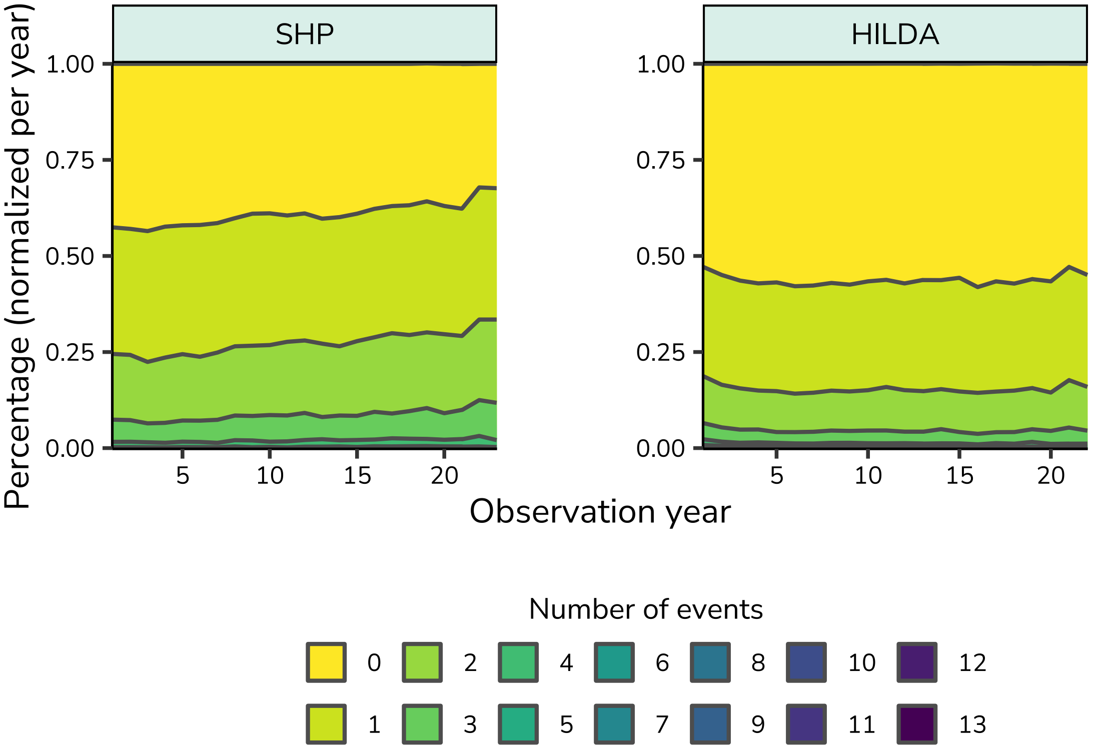

Note. Source: Swiss Household Panel (SHP) and Household, Income and Labour Dynamics in Australia (HILDA).

Supplementary Fig. S11: Contemporaneous joint probability of all event combinations (SHP).

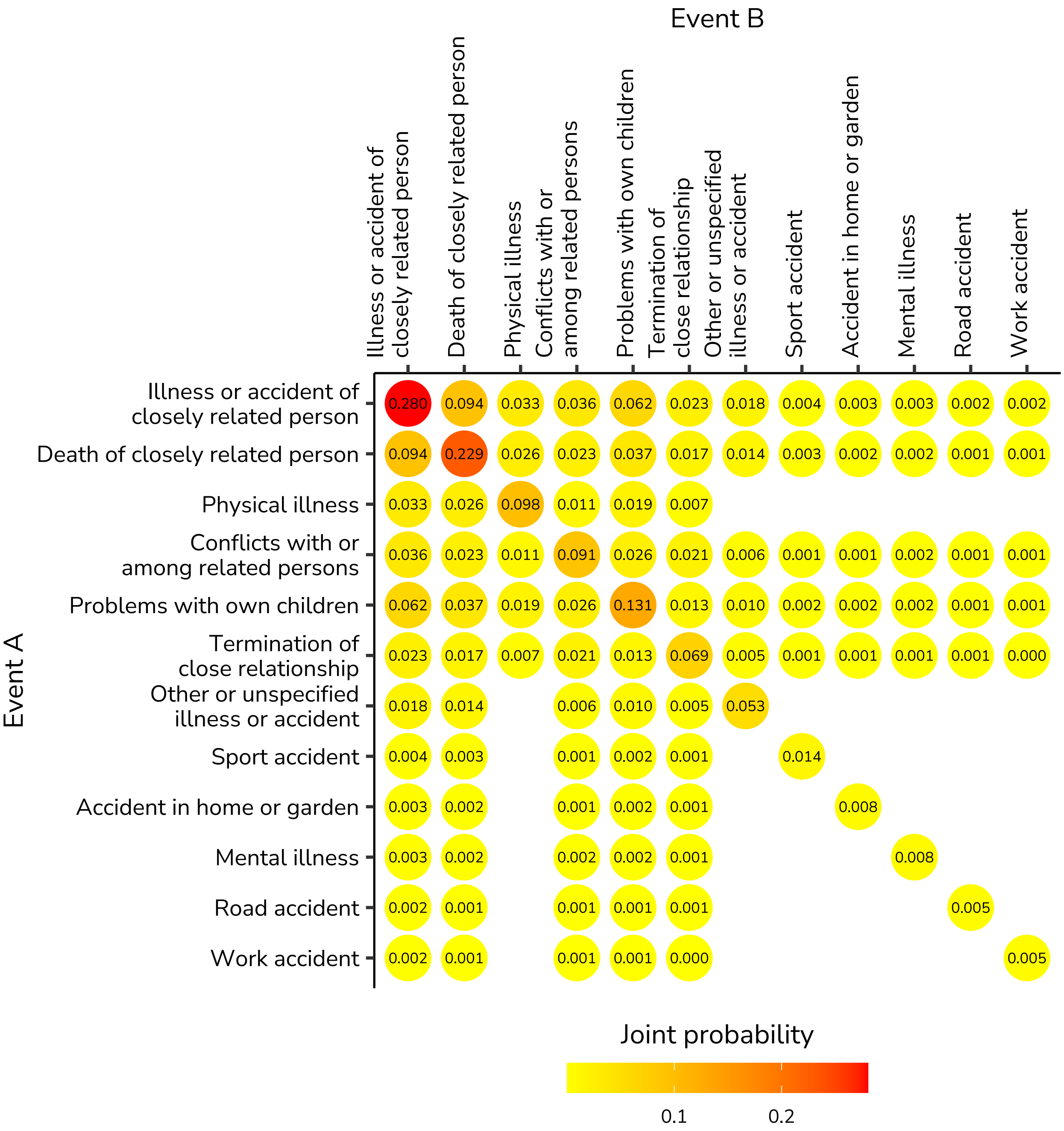

Note. Source: Swiss Household Panel (SHP).

Supplementary Fig. S12: Lag-1 joint probability of all event combinations (SHP).

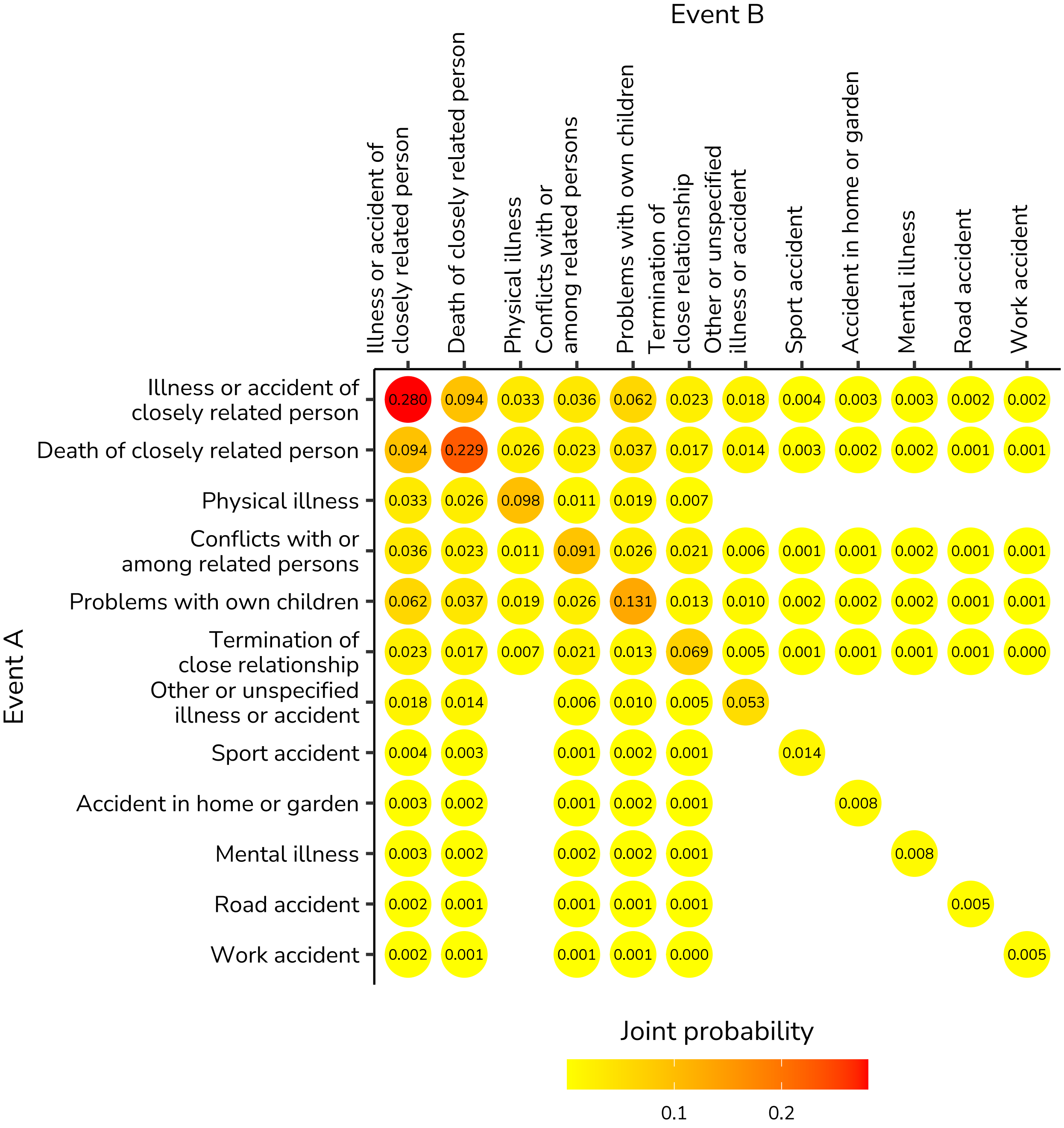

Event A \ Event B	Illness or accident of closely related person	Death of closely related person	Physical illness	Conflicts with or among related persons	Problems with own children	Termination of close relationship	Other or unspecified illness or accident	Sport accident	Accident in home or garden	Mental illness	Road accident	Work accident
Illness or accident of closely related person	0.280	0.094	0.033	0.036	0.062	0.023	0.018	0.004	0.003	0.003	0.002	0.002
Death of closely related person	0.094	0.229	0.026	0.023	0.037	0.017	0.014	0.003	0.002	0.002	0.001	0.001
Physical illness	0.033	0.026	0.098	0.011	0.019	0.007						
Conflicts with or among related persons	0.036	0.023	0.011	0.091	0.026	0.021	0.006	0.001	0.001	0.002	0.001	0.001
Problems with own children	0.062	0.037	0.019	0.026	0.131	0.013	0.010	0.002	0.002	0.002	0.001	0.001
Termination of close relationship	0.023	0.017	0.007	0.021	0.013	0.069	0.005	0.001	0.001	0.001	0.001	0.000
Other or unspecified illness or accident	0.018	0.014		0.006	0.010	0.005	0.053					
Sport accident	0.004	0.003		0.001	0.002	0.001		0.014				
Accident in home or garden	0.003	0.002		0.001	0.002	0.001			0.008			
Mental illness	0.003	0.002		0.002	0.002	0.001				0.008		
Road accident	0.002	0.001		0.001	0.001	0.001					0.005	
Work accident	0.002	0.001		0.001	0.001	0.000						0.005

Note. Source: Swiss Household Panel (SHP).

Supplementary Fig. S13: Contemporaneous joint probability of all event combinations (HILDA).

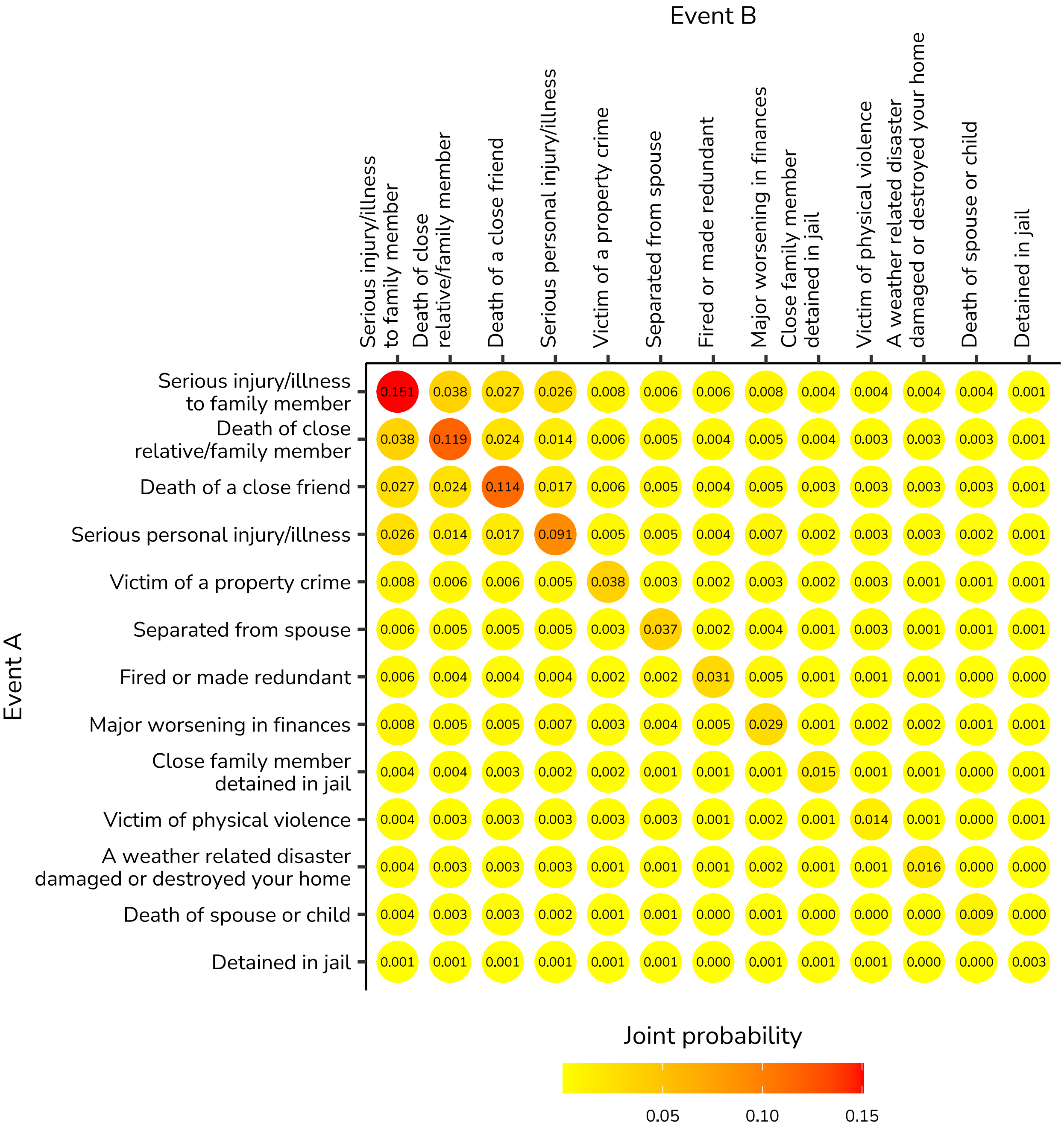

Note. Source: Household, Income and Labour Dynamics in Australia (HILDA). The diagonal reflects the base rates of events.

Supplementary Fig. S14: Lag-1 joint probability of all event combinations (HILDA).

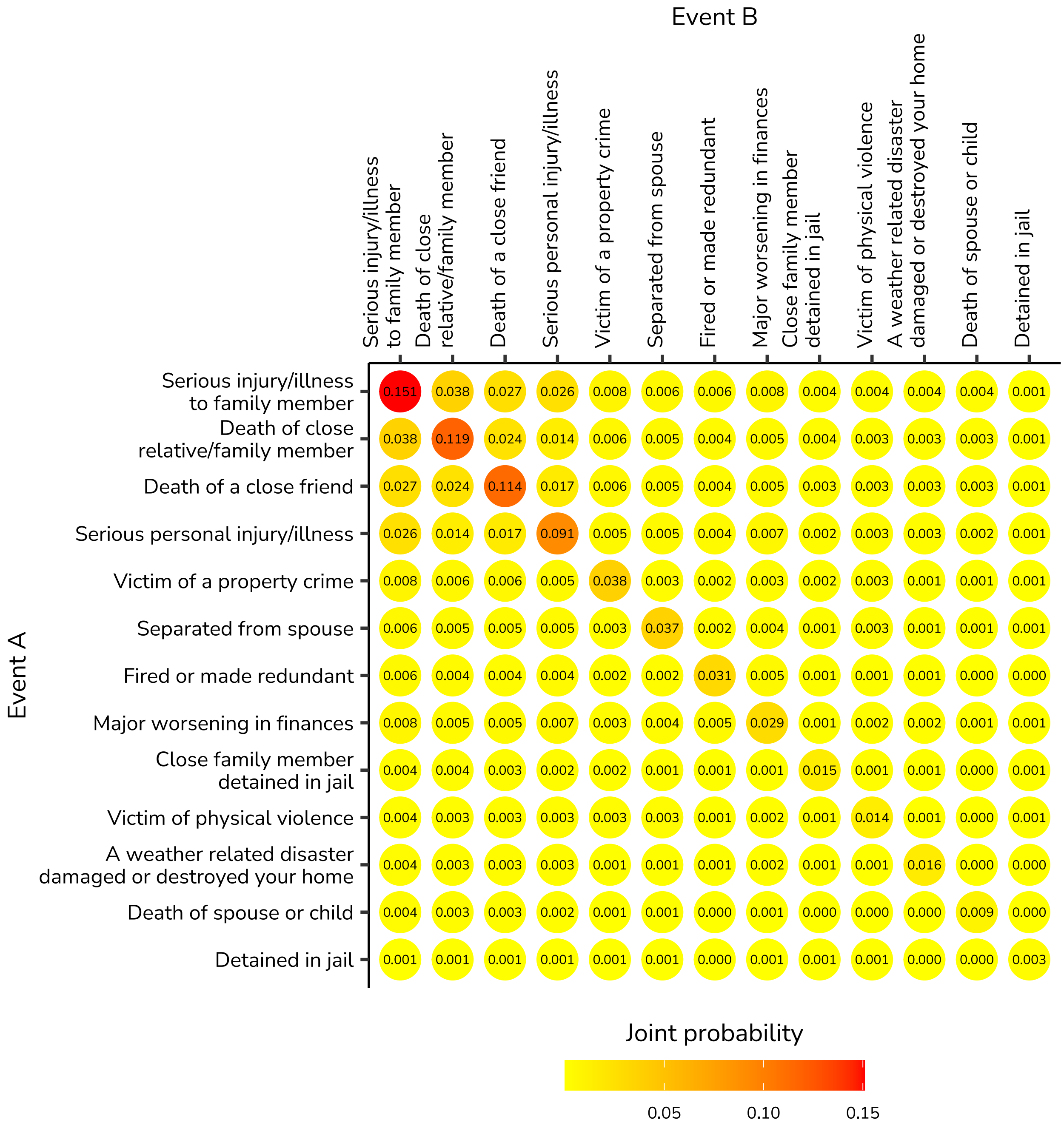

Note. Source: Household, Income and Labour Dynamics in Australia (HILDA).

Supplementary Fig. S15: Contemporaneous conditional probability of all event combinations (SHP).

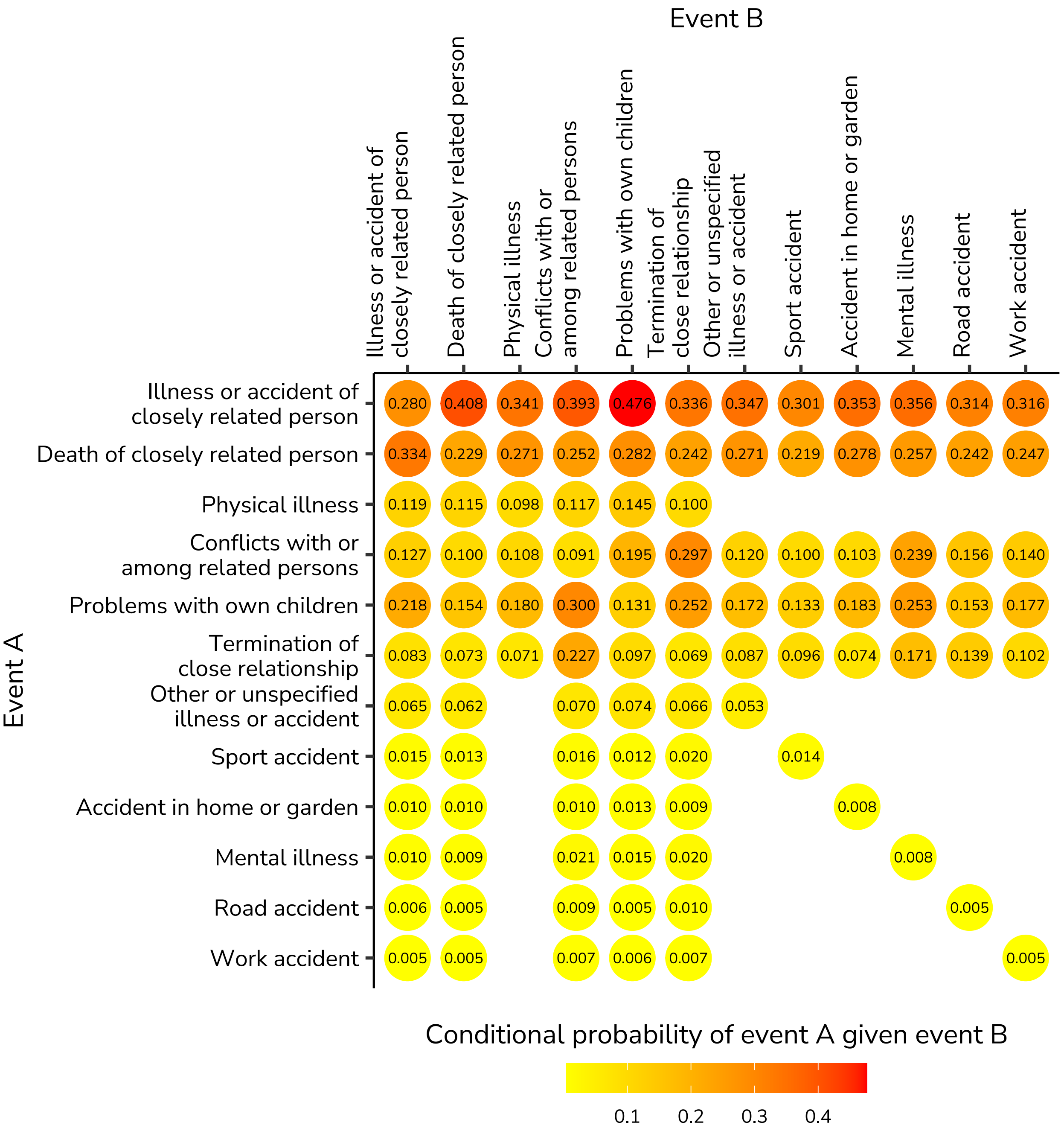

Note. Source: Swiss Household Panel (SHP). The conditional probability indicates the probability of event A (rows) given that event B (columns) happened, ranging from low (yellow) to high (red).

Supplementary Fig. S16: Lag-1 conditional probability of all event combinations (SHP).

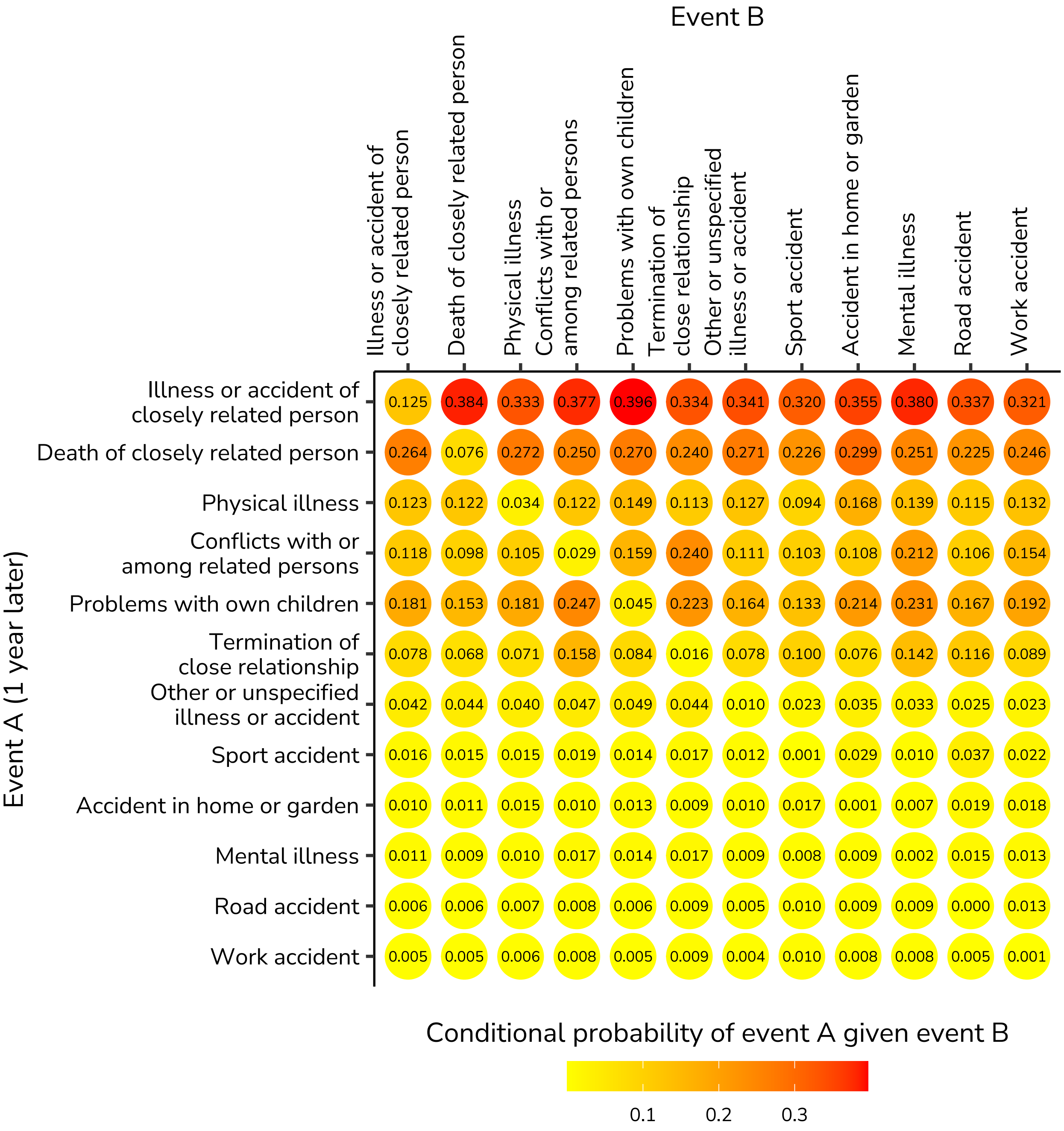

Note. Source: Swiss Household Panel (SHP). The conditional probability indicates the probability of event A (rows) given that event B (columns) happened the year prior, ranging from low (yellow) to high (red).

Supplementary Fig. S17: Contemporaneous conditional probability of all event combinations (HILDA).

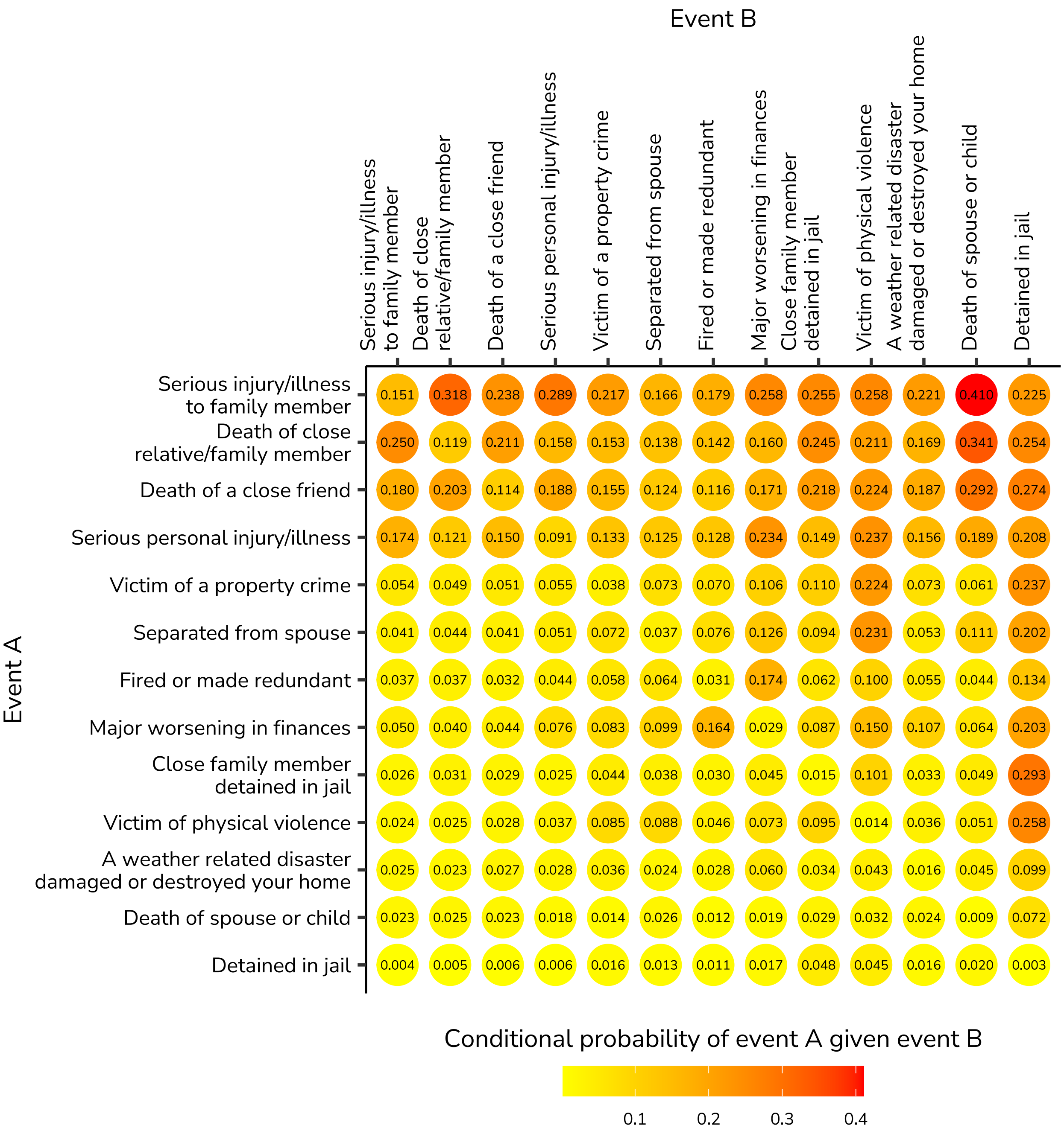

Note. Source: Household, Income and Labour Dynamics in Australia (HILDA). The conditional probability indicates the probability of event A (rows) given that event B (columns) happened, ranging from low (yellow) to high (red).

Supplementary Fig. S18: Lag-1 conditional probability of all event combinations (HILDA).

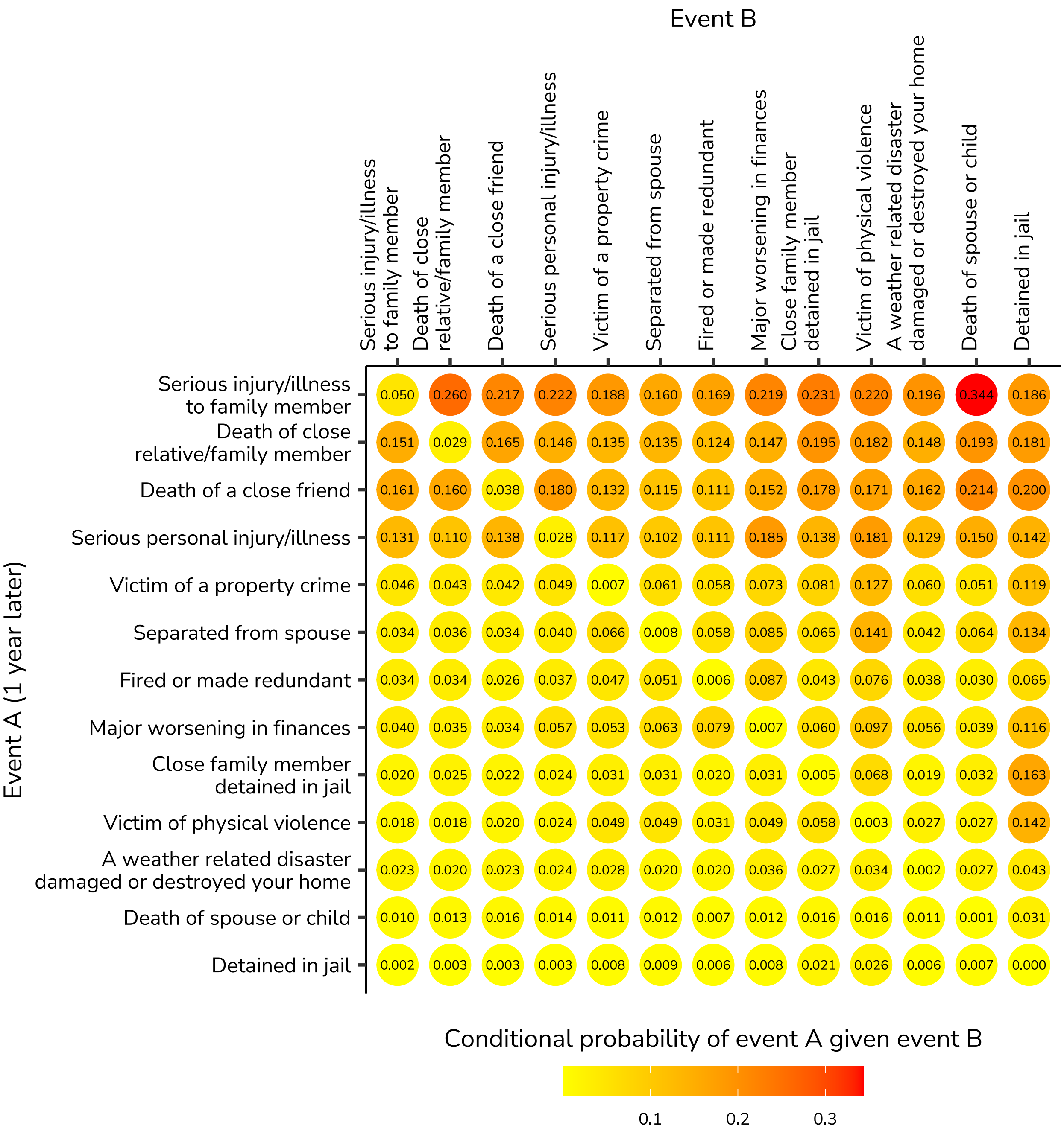

Note. Source: Household, Income and Labour Dynamics in Australia (HILDA). The conditional probability indicates the probability of event A (rows) given that event B (columns) happened the year prior, ranging from low (yellow) to high (red).

Supplementary Table S5: Estimates of the Poisson, frailty, and Polya urn models in the accumulation analysis across 10 years.

	Poisson	Frailty					Polya urn					
	Fixed	Fixed	Random σ		Fit (Poisson-Frailty)		Fixed		Random σ		Fit (Frailty-Polya)	
	λ	λ	i	h	ΔAIC	ΔBIC	p	ϕ	i	h	ΔAIC	ΔBIC
SHP	0.90	0.79	0.41	0.30	3798.02	3781.08	0.13	445.08	0.37	0.37	606.54	598.07
	[0.89,	[0.78,	[0.38,	[0.27,			[0.13,		[0.33,	[0.33,		
	0.91]	0.81]	0.44]	0.34]			0.14]		0.42]	0.42]		
HILDA	0.62	0.51	0.49	0.40	9579.56	9560.95	0.04	118	0.45	0.45	173.78	164.48
	[0.61,	[0.50,	[0.47,	[0.38,			[0.04,	783	[0.43,	[0.43,		
	0.62]	0.52]	0.51]	0.43]			0.04]	4.05	0.47]	0.47]		

Note. Estimates are on the natural scale (profile 95% profile confidence intervals in brackets). Fit comparisons subtract the fit of the model on the right from that of the model on the left, such that a positive number indicates the model on the left fits better. λ = Yearly rate of adverse life events; p = Per-trial probability of adverse life events; ϕ = Dispersion; i = Individual; h = Household; AIC = Akaike Information Criterion; BIC = Bayesian Information Criterion. Source: Swiss Household Panel (SHP) and Household, Income and Labour Dynamics in Australia Survey (HILDA).

Supplementary Table S6: Estimates of the Poisson, frailty, and Polya urn models in the accumulation analysis across 15 years.

	Poisson	Frailty					Polya urn					
	Fixed	Fixed	Random σ		Fit (Poisson-Frailty)		Fixed		Random σ		Fit (Frailty-Polya)	
	λ	λ	i	h	ΔAIC	ΔBIC	p	ϕ	i	h	ΔAIC	ΔBIC
SHP	0.94	0.84	0.38	0.33	5005.78	4988.62	0.14	295.13	0.40	0.40	731.27	722.69
	[0.93,	[0.82,	[0.35,	[0.29,			[0.14,		[0.36,	[0.36,		
	0.95]	0.86]	0.41]	0.37]			0.14]		0.45]	0.45]		
HILDA	0.62	0.52	0.48	0.38	9137.14	9118.69	0.04	217	0.42	0.42	147.94	138.72
	[0.62,	[0.51,	[0.46,	[0.35,			[0.04,	4.71	[0.40,	[0.40,		
	0.63]	0.53]	0.50]	0.40]			0.04]		0.44]	0.44]		

Note. Estimates are on the natural scale (profile 95% profile confidence intervals in brackets). Fit comparisons subtract the fit of the model on the right from that of the model on the left, such that a positive number indicates the model on the left fits better. λ = Yearly rate of adverse life events; p = Per-trial probability of adverse life events; ϕ = Dispersion; i = Individual; h = Household; AIC = Akaike Information Criterion; BIC = Bayesian Information Criterion. Source: Swiss Household Panel (SHP) and Household, Income and Labour Dynamics in Australia Survey (HILDA).

Supplementary Table S7: Model estimates of distributions fit to twenty-year cumulative adverse life event counts.

	Poisson		Exponential		Log-normal			Power-law		Log-Likelihood Ratio cf. Log-normal		
	x_{min}	λ	x_{min}	λ	x_{min}	μ	σ	x_{min}	α	Poisson	Exponential	Power-law
SHP	36	40.27	31	0.14	24	3.29	0.31	31	6.00	2.70 ($p = 0.01$)	1.17 ($p = 0.24$)	2.97 ($p < .01$)
HILDA	42	51.61	13	0.13	10	2.47	0.55	26	5.16	4.21 ($p < .01$)	1.59 ($p = 0.11$)	3.11 ($p < .01$)

Note. The log-likelihood ratio compares the fit of the log-normal model to an alternative model (Poisson, exponential, power-law). Positive values indicate the log-normal model fits better, whereas negative values favour the alternative model. The two-sided p-value tests whether the difference in fit is statistically significant. Source: Swiss Household Panel (SHP) and Household, Income and Labour Dynamics in Australia Survey (HILDA).

Supplementary Fig. S19: Range of the accumulation of adverse life events across twenty consecutive years.

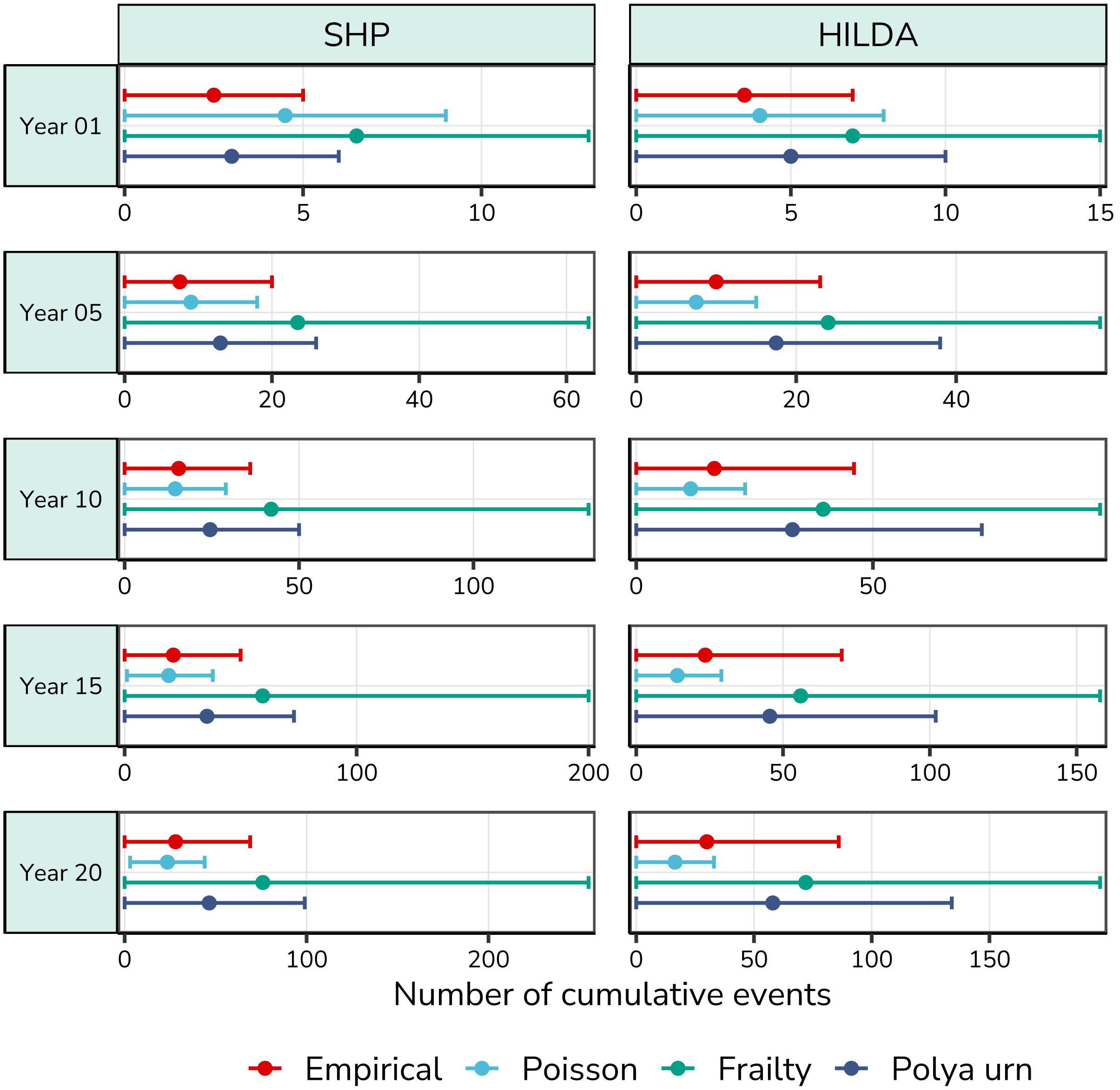

Note. Accumulation analysis based on a sample of 1,370 individuals and 1,134 households (Source: Swiss Household Panel, SHP) and 3,700 individuals and 8,628 households (Source: Household, Income and Labour Dynamics in Australia, HILDA). The empirical data is shown in red dots, with the fit of the Poisson, frailty, and Polya urn model shown in blue, green, and purple, respectively (1,000 model simulations). The horizontal lines extend from the minimum to the maximum number of adverse life events, indicating the median with a dot. Particularly the frailty model predicts a much heavier tail than empirically observed.

Supplementary Fig. S20: Fit of heavy-tailed distributions to twenty-year cumulative adverse life event counts.

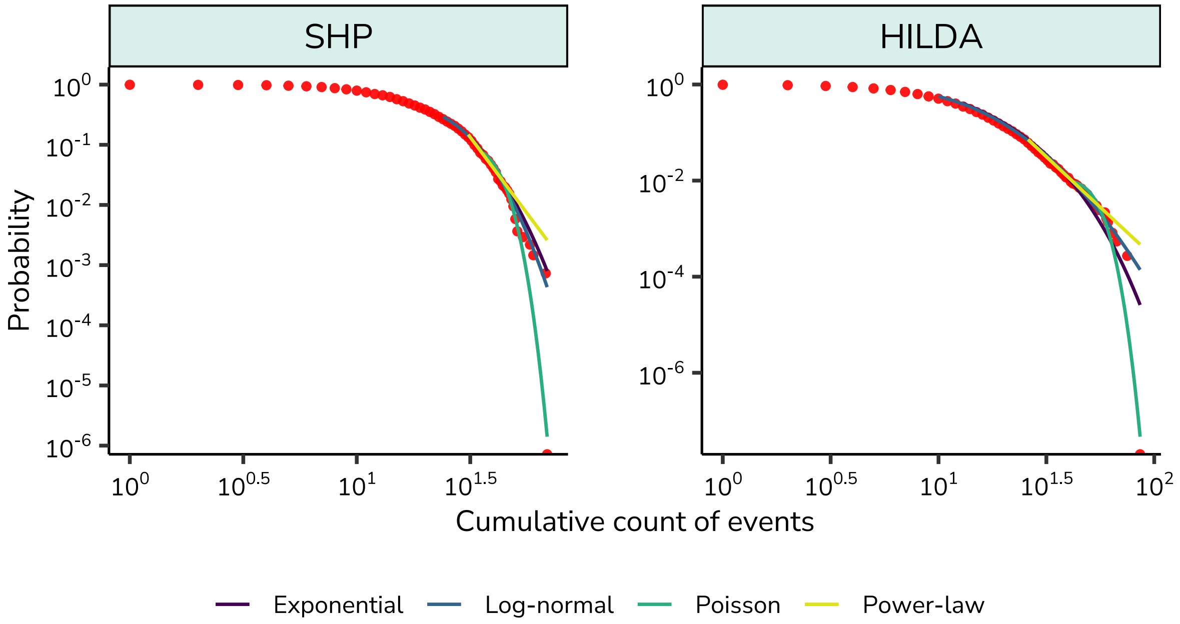

Note. Red dots indicate empirical data. The exponential and log-normal distribution appear to offer the best fit for both datasets as indicated by the log-likelihood ratio (Table S7;). Source: Swiss Household Panel (SHP) and Household, Income and Labour Dynamics in Australia Survey (HILDA).
